# Supplementary material for: Proteomics Identifies Substrates and a Novel Component in hSnd2-Dependent ER Protein Targeting
Source: Cells. 2022 Sep 19;11(18):2925. doi: 10.3390/cells11182925 (PMC9496750; doi:10.3390/cells11182925)
Supplement: Supplementary file 1 [file cells-11-02925-s001.zip › cells-1904948-supplementary.pdf]

**Supplemental Information**

# Proteomics identifies substrates and a novel component in hSnd2-dependent protein targeting to the human ER

**Andrea Tirincsi, Sarah O´Keefe, Duy Nguyen, Mark Sicking, Johanna Dudek, Friedrich Förster, Martin Jung, Drazena Hadzibeganovic, Volkhard Helms, Stephen High, Richard Zimmermann and Sven Lang**

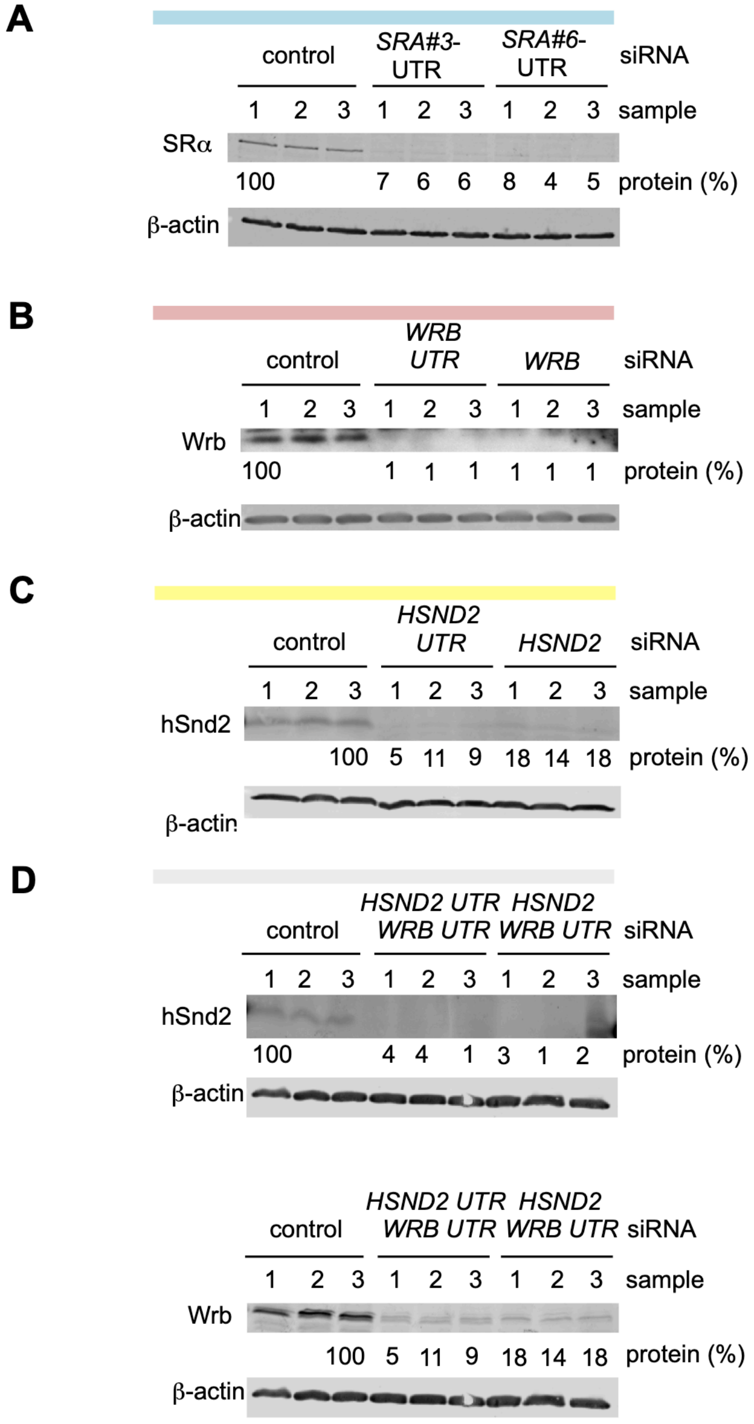
A
control
SRA#3-UTR
SRA#6-UTR
siRNA
1 2 3 1 2 3 1 2 3 sample
SRα
100 7 6 6 8 4 5 protein (%)
β-actin
B
control
WRB UTR
WRB
siRNA
1 2 3 1 2 3 1 2 3 sample
Wrb
100 1 1 1 1 1 1 protein (%)
β-actin
C
control
HSND2 UTR
HSND2
siRNA
1 2 3 1 2 3 1 2 3 sample
hSnd2
100 5 11 9 18 14 18 protein (%)
β-actin
D
control
HSND2 UTR WRB UTR
HSND2 WRB UTR
siRNA
1 2 3 1 2 3 1 2 3 sample
hSnd2
100 4 4 1 3 1 2 protein (%)
β-actin
control
HSND2 UTR WRB UTR
HSND2 WRB UTR
siRNA
1 2 3 1 2 3 1 2 3 sample
Wrb
100 5 11 9 18 14 18 protein (%)
β-actin

**Figure S1. Western blots confirming depletion of various components for protein targeting to the ER by quantitative MS in HeLa cells. Related to Figures 1 and 3.** (A-D) The experimental strategy in the MS experiments involved siRNA-mediated gene silencing using two different siRNAs for each target and one non-targeting (control) siRNA, respectively with three replicates for each siRNA, label-free quantitative proteomic analysis and differential protein abundance analysis to identify negatively affected proteins (i.e. clients) as well as positively affected proteins (i.e. compensatory mechanisms). Knock-down efficiencies were evaluated by Western blot. Only the respective areas of interest are shown. Results are presented as % of residual protein levels (normalized to ß-actin) relative to control, which was set to 100%. (A**)** *SRA* silencing; (B) *WRB* silencing; (C) *HSND2* silencing; (D) simultaneous *HSND2* plus *WRB* silencing. Notably, the same blot was probed with antibodies against hSnd2, Wrb, and ß-actin respectively, and therefore the same loading control is shown twice. The corresponding original Western blots are shown in Figures S2 and S3, respectively.

Original Western blots confirming SRA silencing shown in Fig. 1A and S1A, where indicated pancreatic rough microsomes (RM) served for identification

SRα

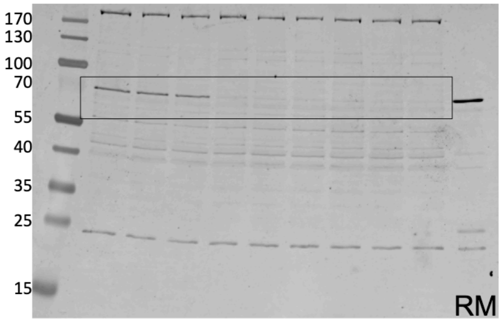


ß-actin

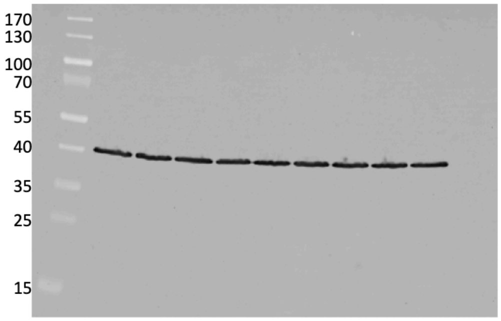


Original  Western blots confirming WRB silencing shown in Fig. 1B and S1B, where indicated pancreatic rough microsomes (RM) served for identification, guided by prestained molecular mass markers the PVDF membrane was cut into pieces

WRB

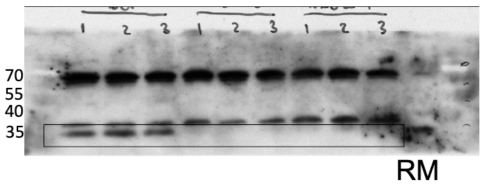


ß-actin

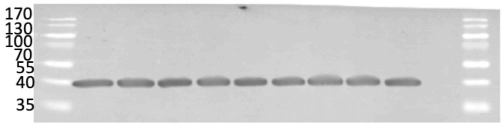

**Figure S2. Original Western blots confirming depletion of various components for protein targeting to the ER by quantitative MS in HeLa cells. Related to Figures 1 and S1.** The experimental strategy in the MS experiments involved siRNA-mediated gene silencing using two different siRNAs for each target and one non-targeting (control) siRNA, respectively, with three replicates for each siRNA, label-free quantitative proteomic analysis and differential protein abundance analysis to identify negatively affected proteins (i.e. clients) as well as positively affected proteins (i.e. compensatory mechanisms). Knock-down efficiencies were evaluated by Western blot. Canine pancreatic rough microsomes (on the right of the cropped area) and molecular mass markers (left or left as well as right lane) were loaded to the same gels and served for identification of the protein of interest (cropped area).

Original Western blots confirming HSND2 silencing shown in Fig. 3A and S1C, where indicated pancreatic rough microsomes (RM) served for identification

hSnd2

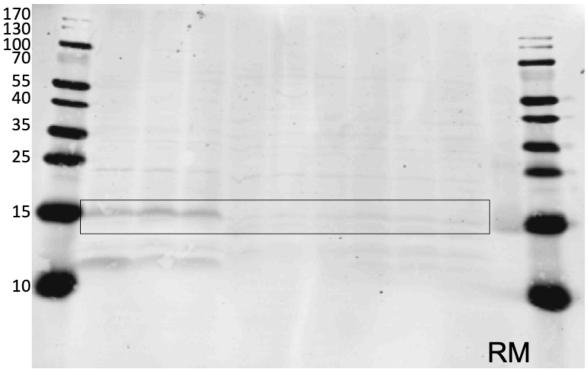


ß-actin

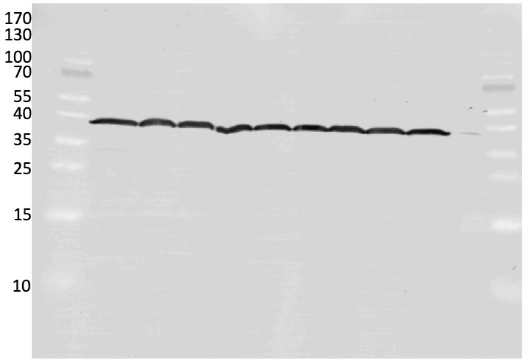


Original Western blots confirming simultaneous silencing of HSND2 + WRB shown in Fig. 3B and S1D where indicated pancreatic rough microsomes (RM) served for identification

hSnd2

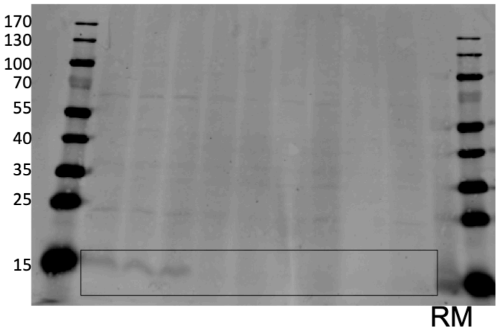


ß-actin

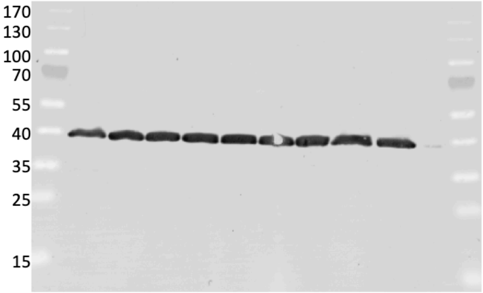


WRB

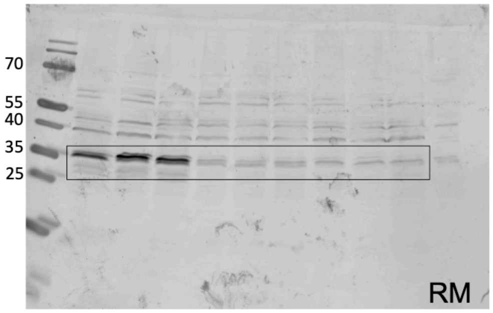


ß-actin

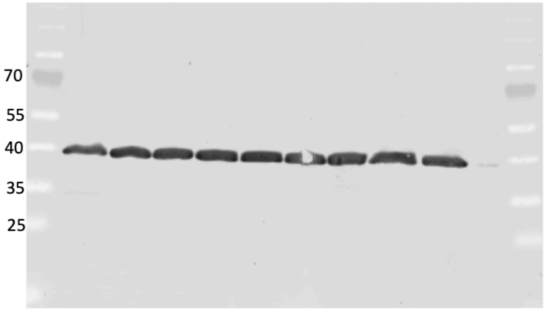

**Figure S3. Original Western blots confirming depletion of various components for protein targeting to the ER by quantitative MS in HeLa cells. Related to Figures 3 and S1.** The experimental strategy in the MS experiments involved siRNA-mediated gene silencing using two different siRNAs for each target and one non-targeting (control) siRNA, respectively, with three replicates for each siRNA, label-free quantitative proteomic analysis and differential protein abundance analysis to identify negatively affected proteins (i.e. clients) as well as positively affected proteins (i.e. compensatory mechanisms). Knock-down efficiencies were evaluated by Western blot. Canine pancreatic rough microsomes (on the right of the cropped area) and molecular mass markers (left or left as well as right lane) were loaded to the same gels and served for identification of the protein of interest (cropped area). Notably, the same blot was probed with antibodies against hSnd2, Wrb, and ß-actin respectively, and therefore the same loading control is shown twice.

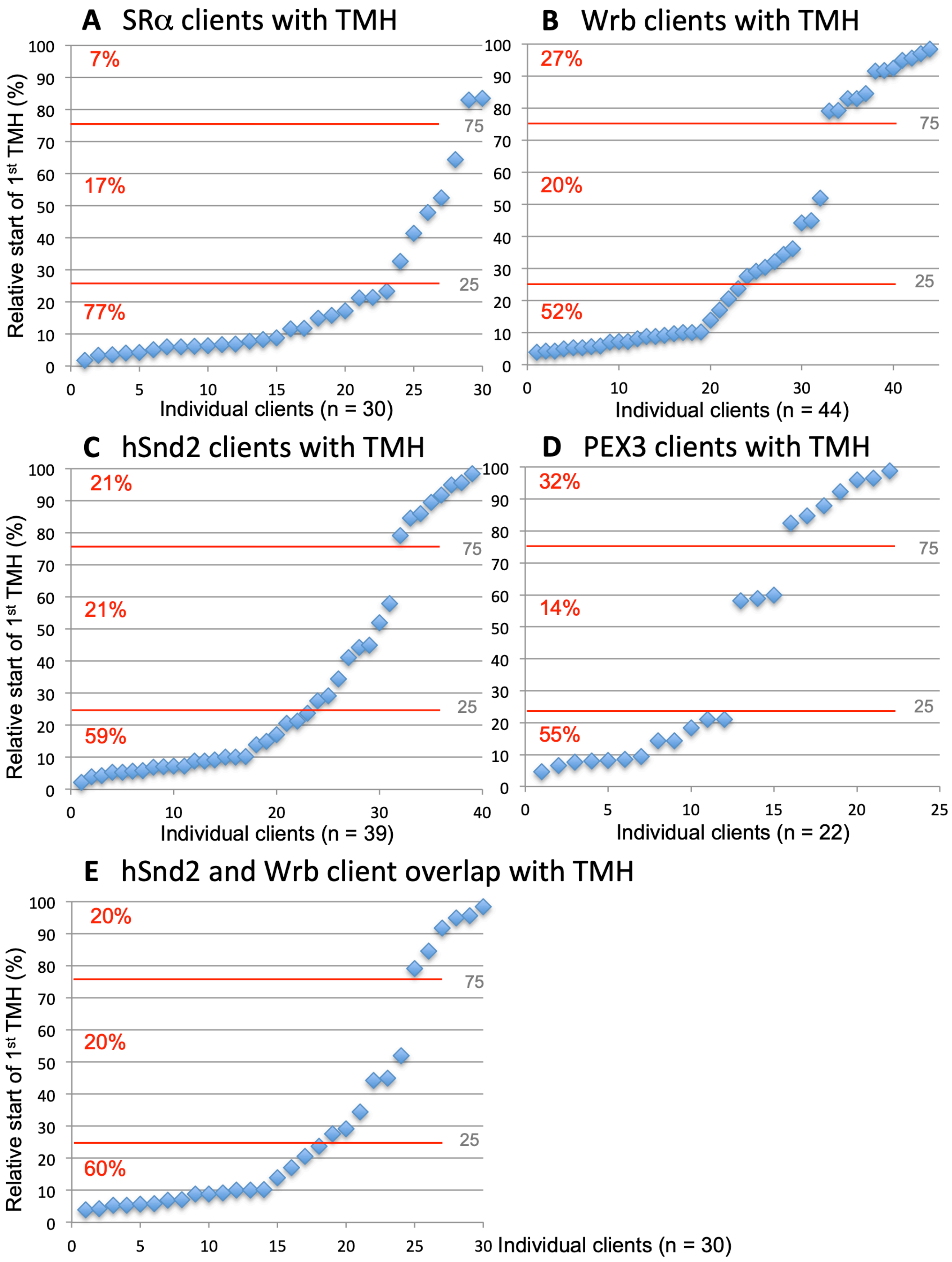
A SRα clients with TMH
7%
17%
77%
75
25
Relative start of 1st TMH (%)
Individual clients (n = 30)
B Wrb clients with TMH
27%
20%
52%
75
25
Individual clients (n = 44)
C hSnd2 clients with TMH
21%
21%
59%
75
25
Relative start of 1st TMH (%)
Individual clients (n = 39)
D PEX3 clients with TMH
32%
14%
55%
75
25
Individual clients (n = 22)
E hSnd2 and Wrb client overlap with TMH
20%
20%
60%
75
25
Relative start of 1st TMH (%)
Individual clients (n = 30)

**Figure S4. Distinguishing Features of SR$\alpha$, Wrb, and hSnd2 Clients with TMH. Related to Figure 2.** (A-D) TMH containing clients were plotted against the location of their first TMH, i.e. position of central amino acid residue of TMH in % of client amino acid residues. For Wrb and hSnd2 clients, the data points and numbers (n) refer to the pooled clients (from the respective single depletion, hSnd2 or Wrb, and the double depletion, hSnd2+Wrb) and are shown in Tables S5 and S6. (E) For comparison, the analysis was also done for the overlap between Wrb and hSnd2 clients. Notably, the uppermost quarter includes membrane proteins with TA, the lowermost quarter those with rather N-terminal TMH.

## SRα clients

**A**

### SP analysis

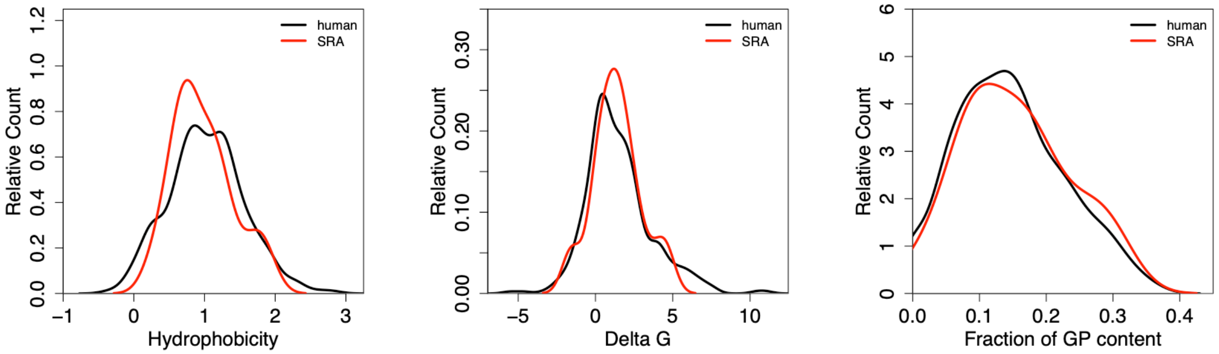


p=0.509 p=0.882 p=0.651

**B**

### SP analysis after segmentation

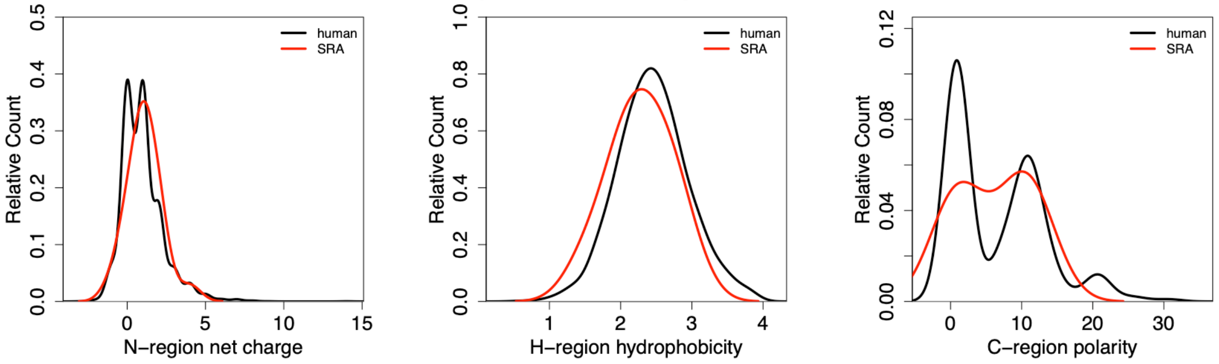


p=0.325 p=0.136 p=0.896

**C**

### TMH analysis

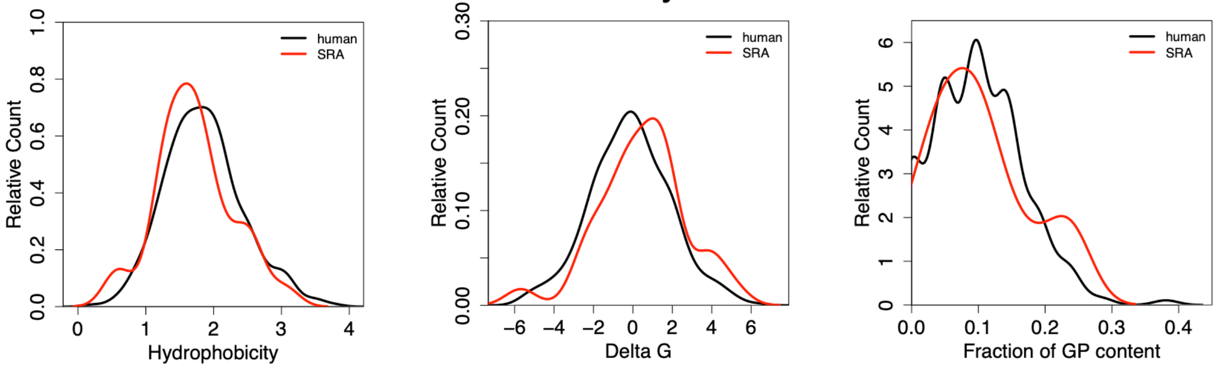


p=0.193 p=0.121 p=0.976

**Figure S5. Physicochemical properties of SP, SP-regions and TMH of SR$\alpha$ clients. Related to Figure 2.** (A,C) Using custom scripts, we computed the hydrophobicity and glycine/proline (GP) content of the sequences of SP (A) or TMH (C). The hydrophobicity score of a peptide was calculated as the averaged hydrophobicity of its amino acids according to the well-known Kyte-Doolittle propensity scale as described [34]. GP content was calculated as the total fraction of glycine and proline in the respective sequence as described [34] . We determined the $\Delta G_{app}$ values of SP and TMH with the $\Delta G_{app}$ predictor for transmembrane helix insertion (http://dgpred.cbr.su.se) and plotted all these values against the relative counts. (B) The properties of N-, H- and C-regions of SP of clients were also analyzed after their segmentation by the Phobius (http://phobius.sbc.su.se) prediction tool. Their properties include total net charge of N-region, hydrophobicity of H-region, and the polarity of C-region. Polarity was calculated as the averaged polarity of its amino acids according to the polarity propensity scale as described [35]. Likewise, hydrophobicity was calculated using the Kyte-Doolittle propensity scale. We also used custom scripts to extract all SP annotations for human proteins from UniProtKB entries and applied the same calculations for all these human SPs (human).

## Wrb clients

**A**

SP analysis

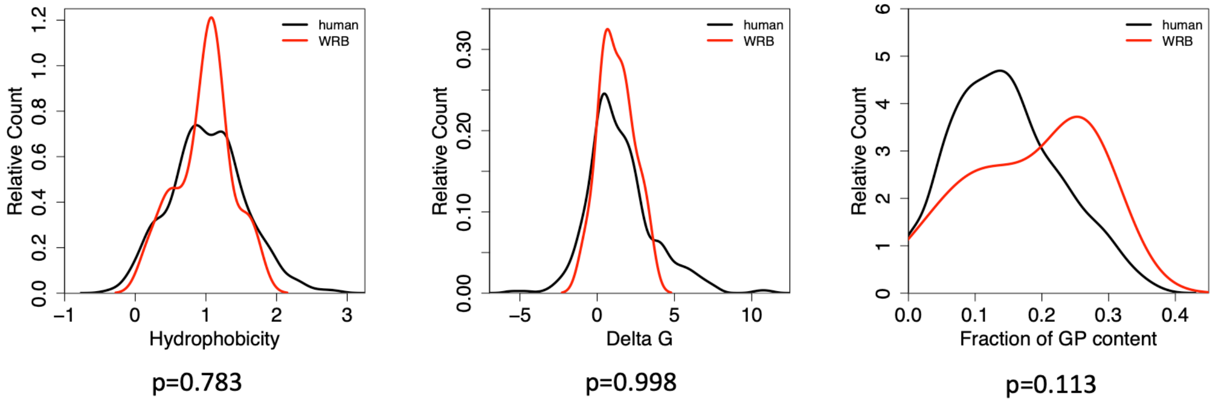


**B**

SP analysis after segmentation

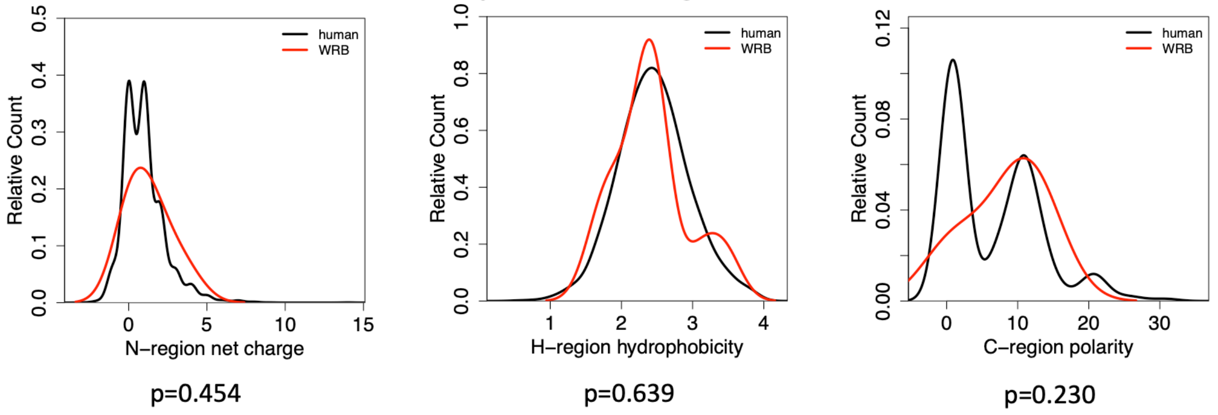


**C**

TMH analysis

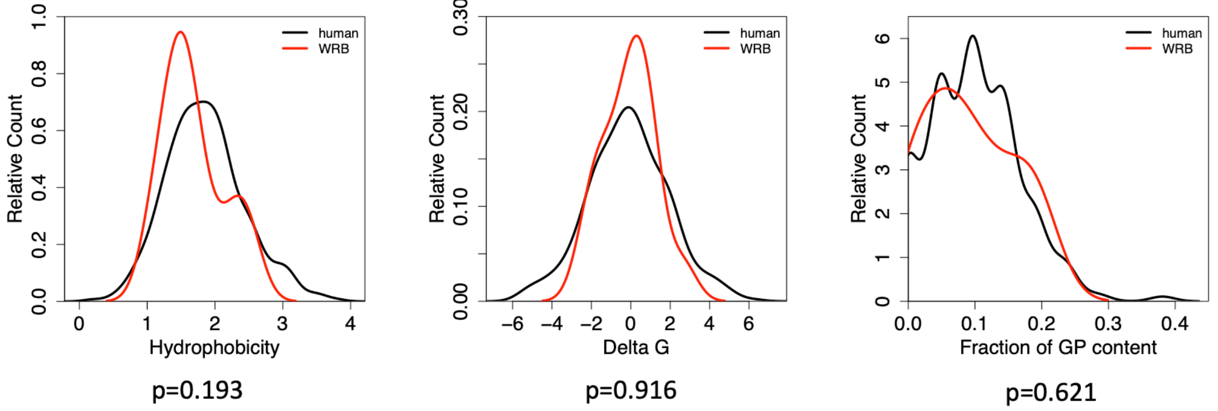

**Figure S6. Physicochemical properties of SP, SP-regions and TMH of Wrb clients. Related to Figure 2.** (A,C) Using custom scripts, we computed the hydrophobicity and glycine/proline (GP) content of the sequences of SP (A) or TMH (C). The hydrophobicity score of a peptide was calculated as the averaged hydrophobicity of its amino acids according to the well-known Kyte-Doolittle propensity scale as described [34]. GP content was calculated as the total fraction of glycine and proline in the respective sequence as described [34]. We determined the $\Delta G_{app}$ values of SP and TMH with the $\Delta G_{app}$ predictor for transmembrane helix insertion (http://dgpred.cbr.su.se) and plotted all these values against the relative counts. (B) The properties of N-, H- and C-regions of SP of clients were also analyzed after their segmentation by the Phobius (http://phobius.sbc.su.se) prediction tool. Their properties include total net charge of N-region, hydrophobicity of H-region, and the polarity of C-region. Polarity was calculated as the averaged polarity of its amino acids according to the polarity propensity scale as described [35]. Likewise, hydrophobicity was calculated using the Kyte-Doolittle propensity scale. We also used custom scripts to extract all SP annotations for human proteins from UniProtKB entries and applied the same calculations for all these human SPs (human).

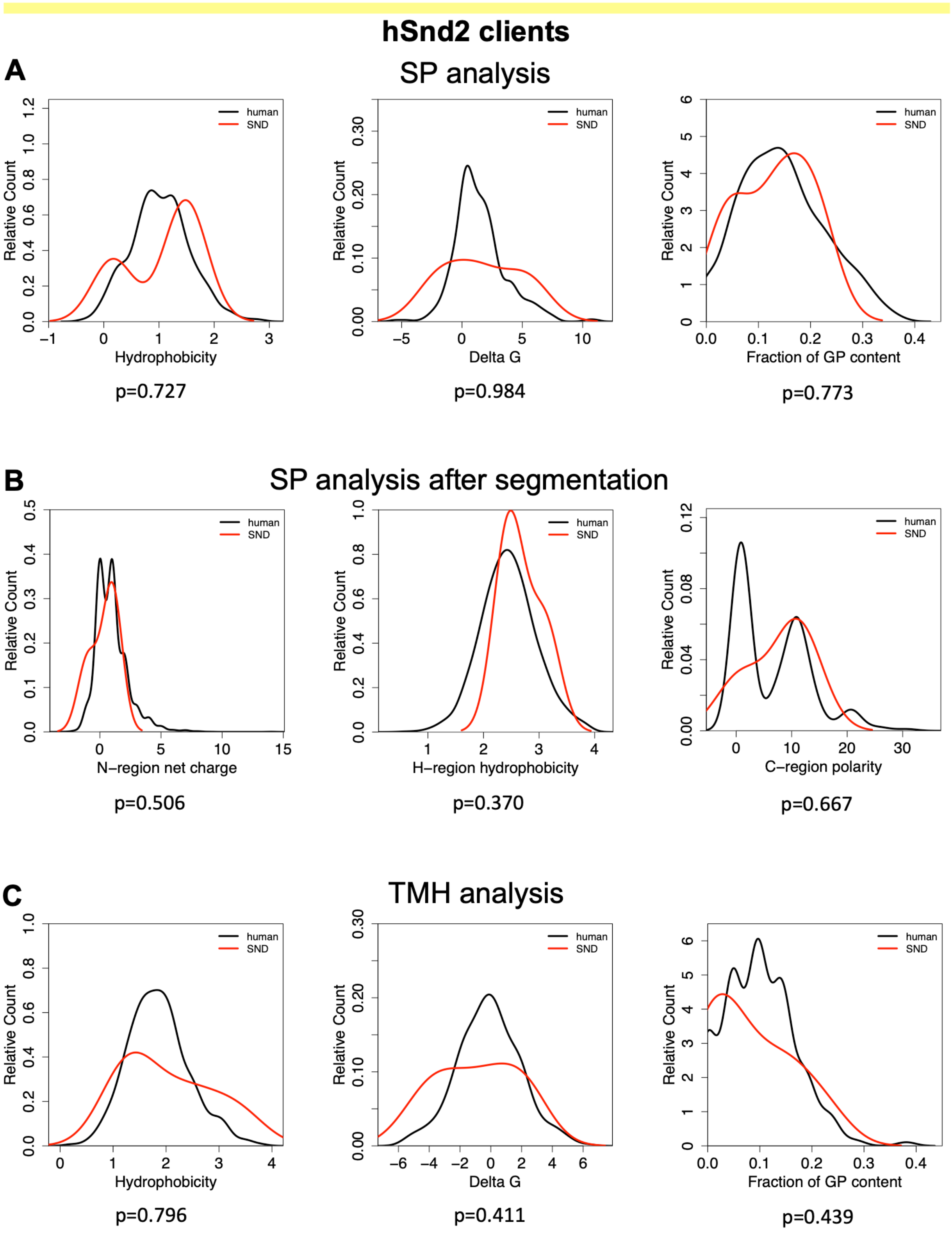
hSnd2 clients
A
SP analysis
Relative Count
Hydrophobicity
p=0.727
Delta G
p=0.984
Fraction of GP content
p=0.773
human
SND
B
SP analysis after segmentation
N−region net charge
p=0.506
H−region hydrophobicity
p=0.370
C−region polarity
p=0.667
C
TMH analysis
Hydrophobicity
p=0.796
Delta G
p=0.411
Fraction of GP content
p=0.439

**Figure S7. Physicochemical properties of SP, SP-regions and TMH of hSnd2 clients. Related to Figure 2.** (A,C) Using custom scripts, we computed the hydrophobicity and glycine/proline (GP) content of the sequences of SP (A) or TMH (C). The hydrophobicity score of a peptide was calculated as the averaged hydrophobicity of its amino acids according to the well-known Kyte-Doolittle propensity scale as described [34]. GP content was calculated as the total fraction of glycine and proline in the respective sequence as described [34]. We determined the $\Delta G_{app}$ values of SP and TMH with the $\Delta G_{app}$ predictor for transmembrane helix insertion (http://dgpred.cbr.su.se) and plotted all these values against the relative counts. (B) The properties of N-, H- and C-regions of SP of clients were also analyzed after their segmentation by the Phobius (http://phobius.sbc.su.se) prediction tool. Their properties include total net charge of N-region, hydrophobicity of H-region, and the polarity of C-region. Polarity was calculated as the averaged polarity of its amino acids according to the polarity propensity scale as described [35]. Likewise, hydrophobicity was calculated using the Kyte-Doolittle propensity scale. We also used custom scripts to extract all SP annotations for human proteins from UniProtKB entries and applied the same calculations for all these human SPs (human).

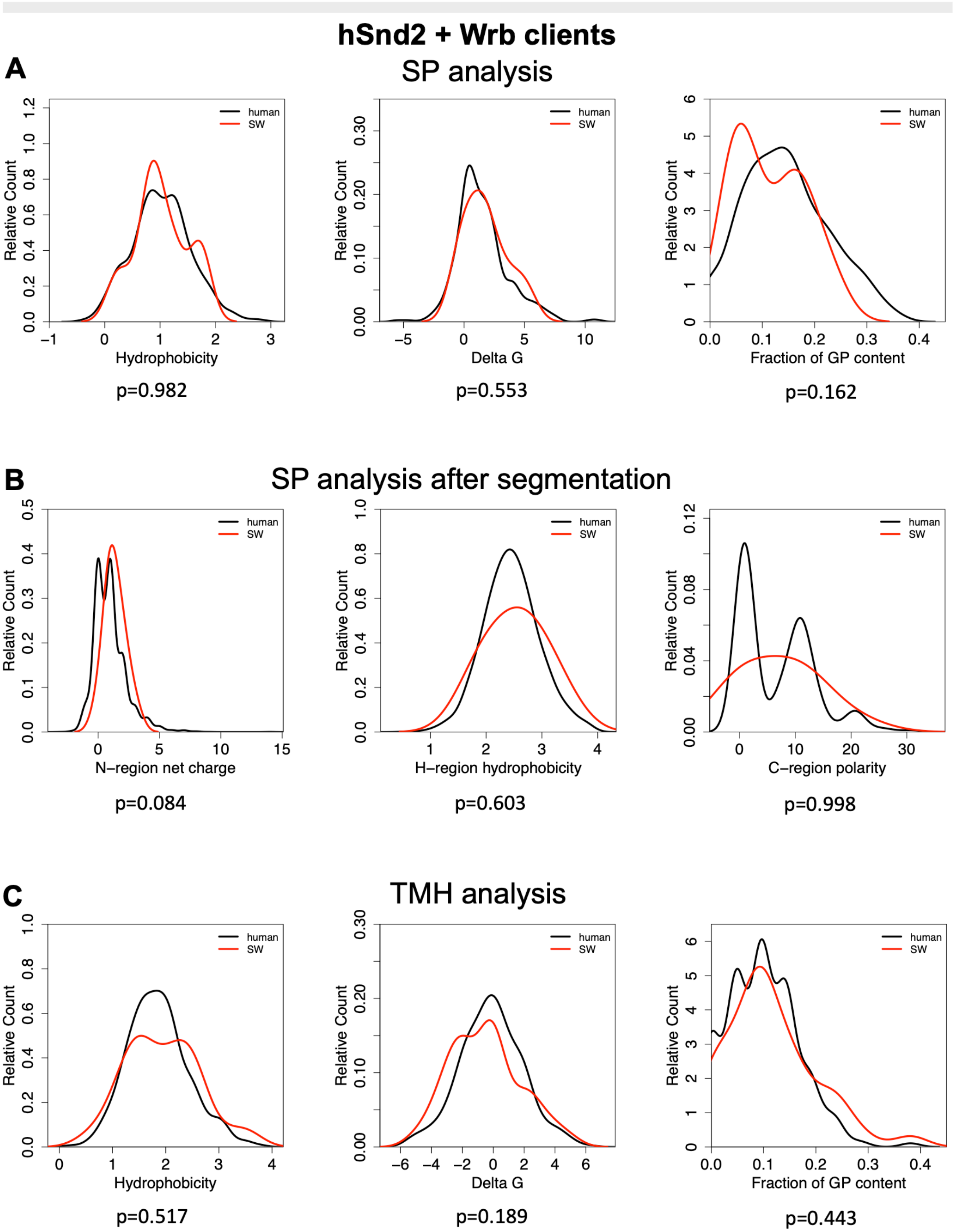
hSnd2 + Wrb clients
A
SP analysis
Relative Count
Hydrophobicity
Delta G
Fraction of GP content
human
SW
p=0.982
p=0.553
p=0.162
B
SP analysis after segmentation
N–region net charge
H–region hydrophobicity
C–region polarity
p=0.084
p=0.603
p=0.998
C
TMH analysis
Hydrophobicity
Delta G
Fraction of GP content
p=0.517
p=0.189
p=0.443

**Figure S8. Physicochemical properties of SP, SP-regions and TMH of hSnd2 plus Wrb clients. Related to Figure 2.** (A,C) Using custom scripts, we computed the hydrophobicity and glycine/proline (GP) content of the sequences of SP (A) or TMH (C). The hydrophobicity score of a peptide was calculated as the averaged hydrophobicity of its amino acids according to the well-known Kyte-Doolittle propensity scale as described [34]. GP content was calculated as the total fraction of glycine and proline in the respective sequence as described [34]. We determined the $\Delta G_{app}$ values of SP and TMH with the $\Delta G_{app}$ predictor for transmembrane helix insertion (http://dgpred.cbr.su.se) and plotted all these values against the relative counts. (B) The properties of N-, H- and C-regions of SP of clients were also analyzed after their segmentation by the Phobius (http://phobius.sbc.su.se) prediction tool. Their properties include total net charge of N-region, hydrophobicity of H-region, and the polarity of C-region. Polarity was calculated as the averaged polarity of its amino acids according to the polarity propensity scale as described [35]. Likewise, hydrophobicity was calculated using the Kyte-Doolittle propensity scale. We also used custom scripts to extract all SP annotations for human proteins from UniProtKB entries and applied the same calculations for all these human SPs (human).

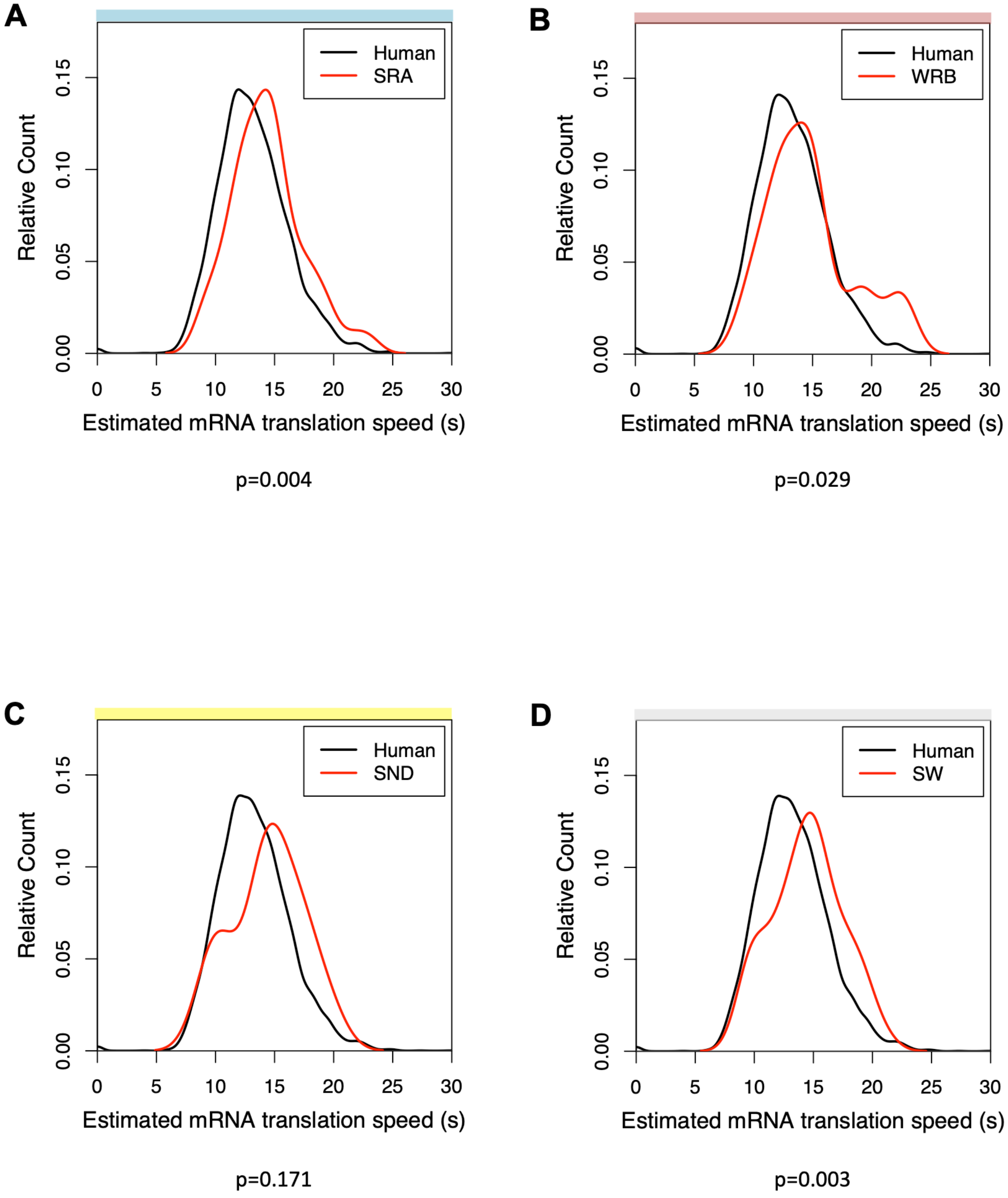

A
Human
SRA
Relative Count
0.00
0.05
0.10
0.15
0
5
10
15
20
25
30
Estimated mRNA translation speed (s)
p=0.004
B
Human
WRB
Relative Count
Estimated mRNA translation speed (s)
p=0.029
C
Human
SND
Relative Count
Estimated mRNA translation speed (s)
p=0.171
D
Human
SW
Relative Count
Estimated mRNA translation speed (s)
p=0.003

**Figure S9. Translation rates of mRNAs coding for clients of SR$\alpha$, Wrb and hSnd2. Related to Figure 2.** (A-D) The full dataset of human mRNA coding sequences (CDS) was downloaded from www.ensemble.org. It contains 110788 CDS records. To avoid a bias in the comparison between the CDS of clients against the full human CDS (background dataset), we only used the CDS transcripts for the proteins identified in the MS data. For this, we translated the full CDS dataset into protein sequences and compared the resulting proteins with the MS data. 4660 (out of 4856) proteins could be matched in this way. The goal of this analysis is to compare the (estimated) translation rates of the mRNAs negatively affected by knock-down which contain either SP or TMH to the estimated translation rates of the mRNAs in the full MS dataset. As we are only interested in the N-terminal part of the sequence, and to ensure comparability of the results independent of the length of the actual mRNA sequences, we always used the first 240 nucleotides (which is equivalent to 80 amino acids) of the CDS for the subsequent analysis. The translation rates were computed based on the codon-specific elongation rates as provided by Trösenmeier et al. [56]. The rates determined there describe the specific elongation speed for each codon during the translation process. Here, the rates were inverted and summed up, resulting in the (estimated) translation speed for the N-terminal 240 nt of each sequence (240 nt. ~ 80 codons). The two distributions were compared using the nonparametric Wilcoxon test.

Original Western blots for siRNA mediated silencing shown in Fig. 4A,
where indicated pancreatic rough microsomes (RM) served for identification.

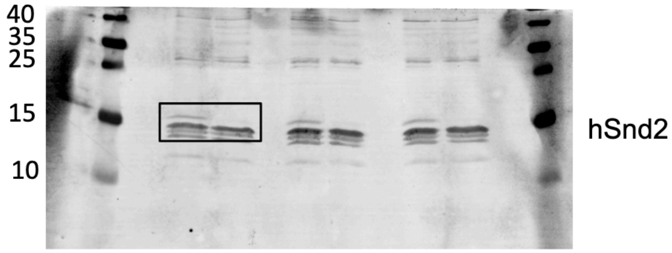


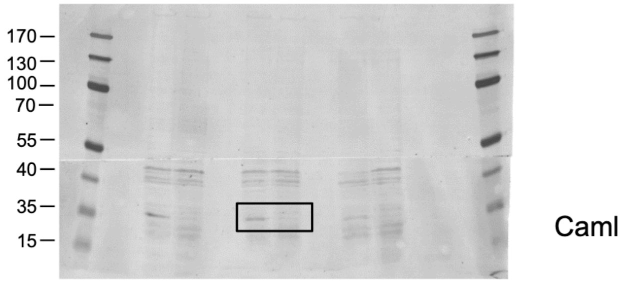


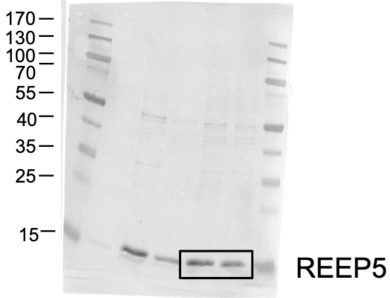


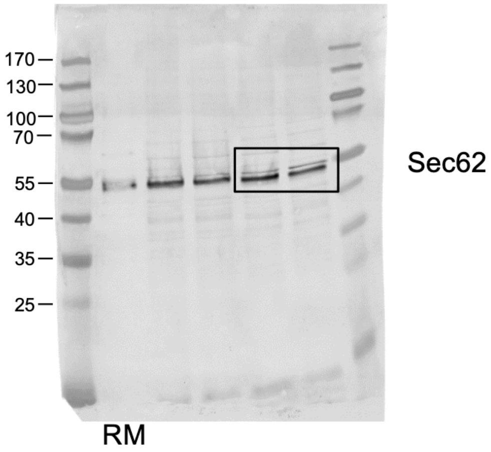


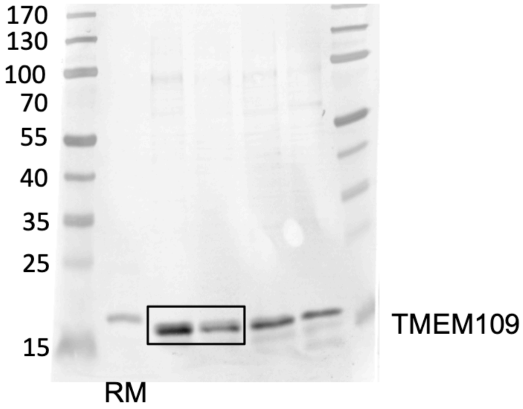

Original Western blots for siRNA mediated silencing shown in Fig. 4A,
where indicated pancreatic rough microsomes (RM) served for identification.

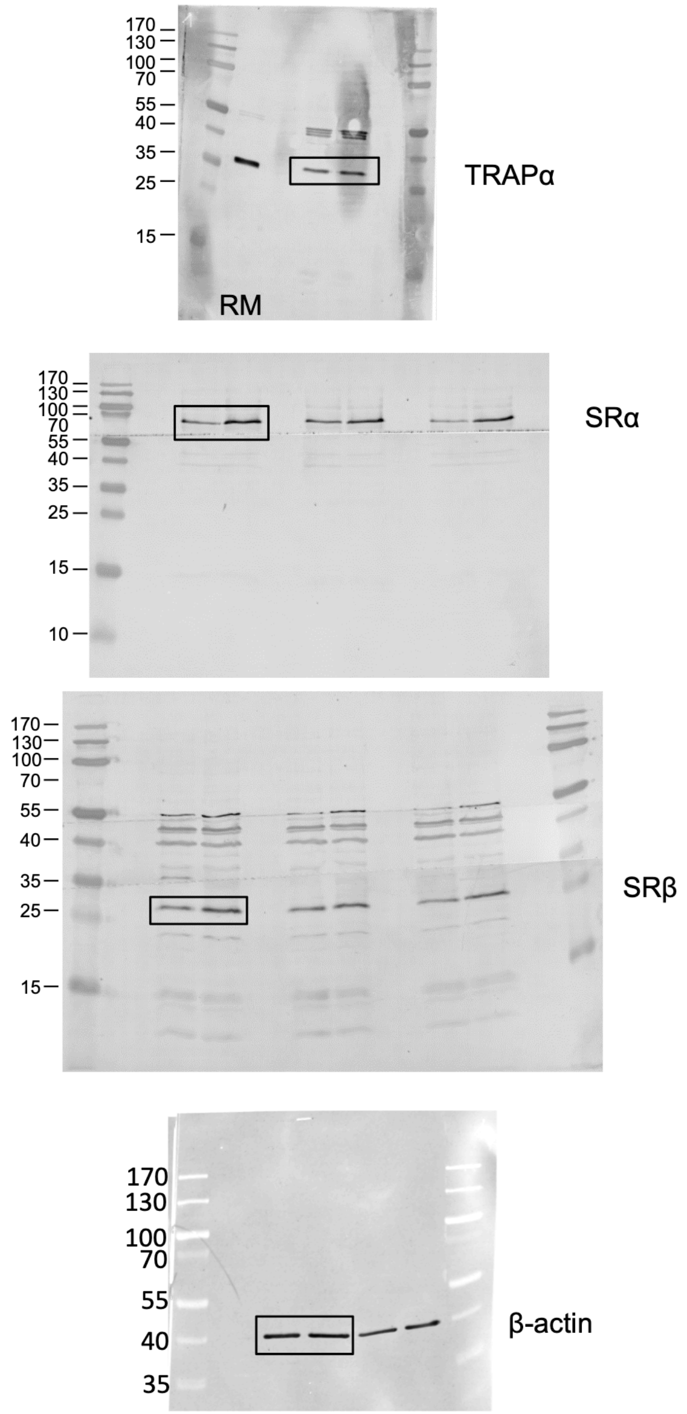

**Figure S10. Original Western blots confirming depletion of various proteins by quantitative MS in HeLa cells. Related to Figure 4.** The experimental strategy was as follows: siRNA-mediated gene silencing using one siRNAs for each target and one non-targeting (control) siRNA, respectively, in at least three independent experiments; knockdown efficiencies as well as a set of putative clients and possible compensatory proteins were evaluated by Western blots. The selection of clients and compensatory components was purely based on the availability of reliable antibodies. For technical reasons, Caml was used as proxy for Wrb. Canine pancreatic rough microsomes (RM, on the left of the cropped area) and molecular mass markers (far left or far left as well as right lane) were loaded to the same gels and served for identification of the protein of interest (cropped area).

Original Western blots for CO-IPs shown in Fig. 5B,
where indicated pancreatic rough microsomes (RM) served for identification,
guided by prestained molecular mass markers the PVDF membrane was cut into pieces

TMEM109

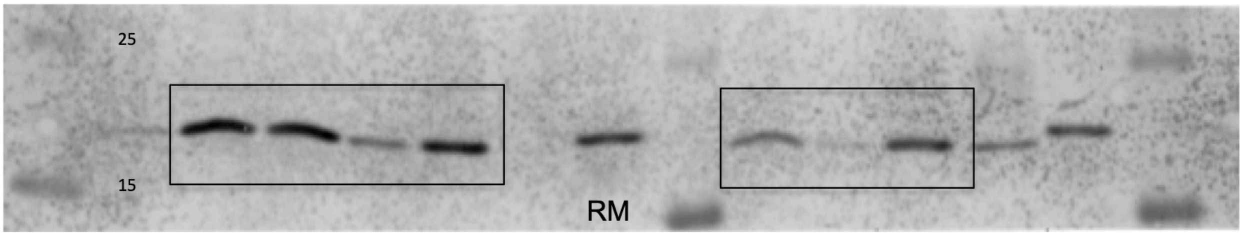


hSnd2

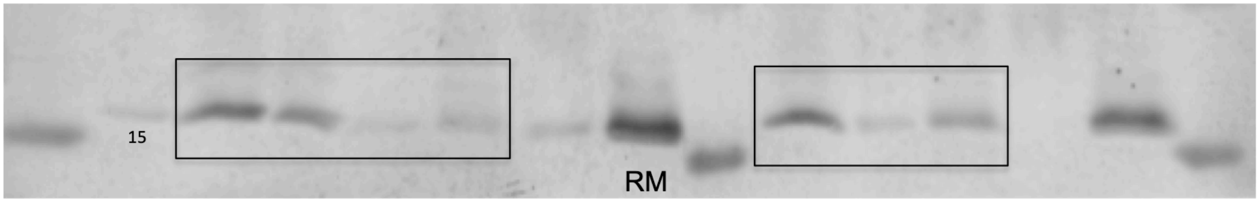


Grp170

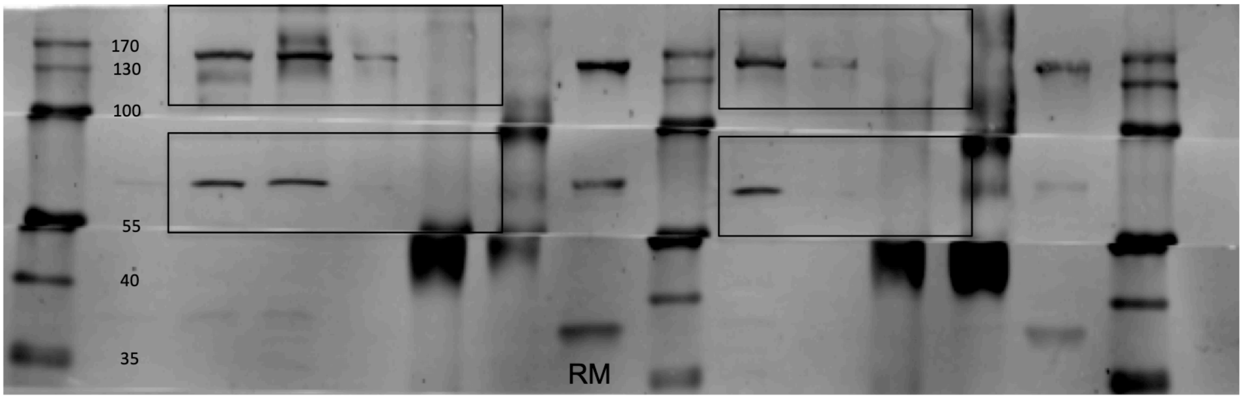


Climp63

**Figure S11. Original Western blots related to Figure 5B.** Canine pancreatic rough microsomes and molecular mass markers were loaded to the same gels and served for identification of the protein of interest (cropped area).

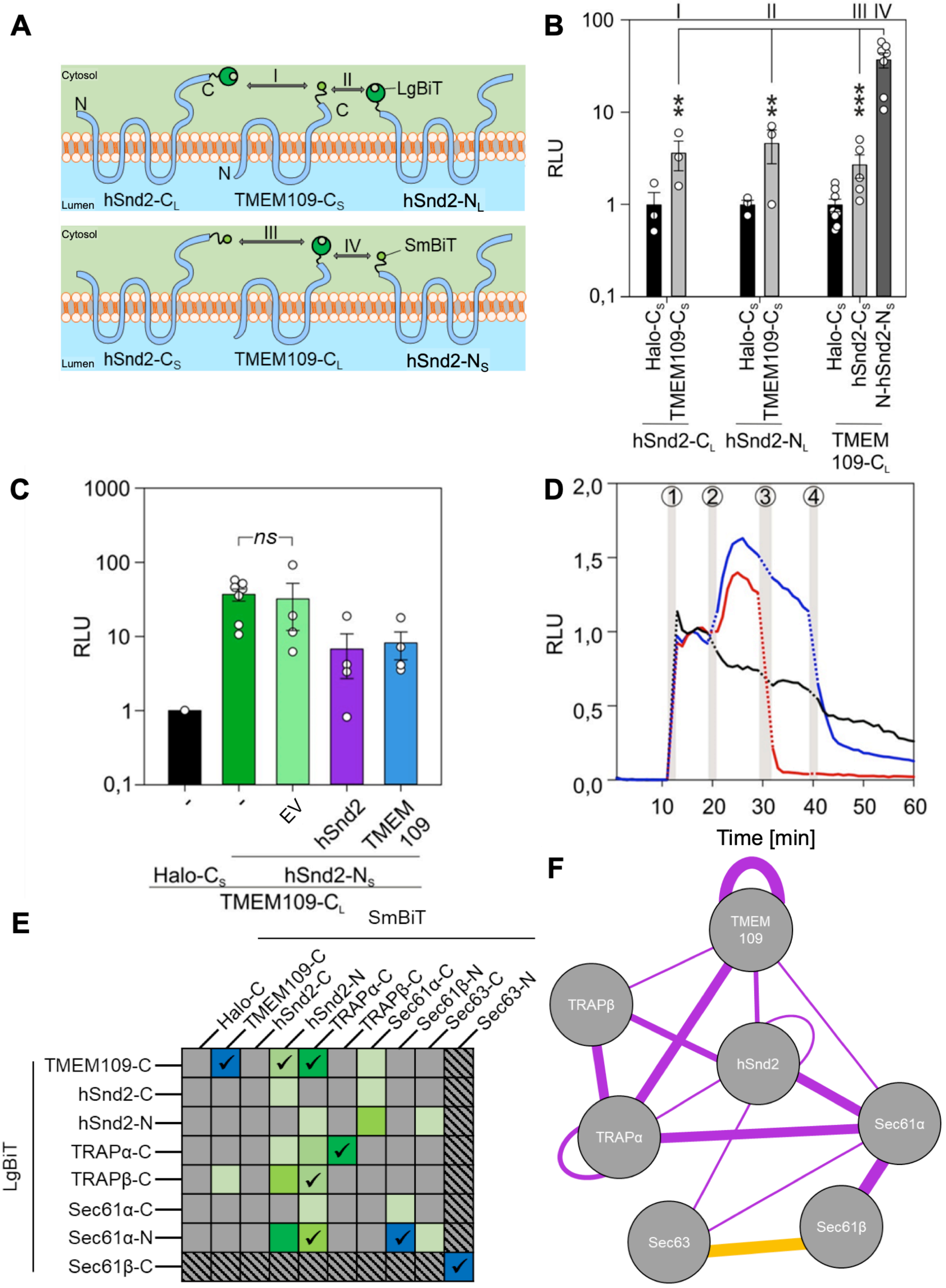
A
Cytosol
Lumen
I
II
III
IV
LgBiT
SmBiT
hSnd2-C$_L$
TMEM109-C$_S$
hSnd2-N$_L$
hSnd2-C$_S$
TMEM109-C$_L$
hSnd2-N$_S$
B
RLU
Halo-C$_S$
TMEM109-C$_S$
hSnd2-C$_S$
N-hSnd2-N$_S$
hSnd2-C$_L$
hSnd2-N$_L$
TMEM 109-C$_L$
C
ns
EV
hSnd2
TMEM 109
Halo-C$_S$
hSnd2-N$_S$
TMEM109-C$_L$
D
Time [min]
E
SmBiT
LgBiT
Halo-C
TMEM109-C
hSnd2-C
hSnd2-N
TRAPα-C
TRAPβ-C
Sec61α-C
Sec61β-N
Sec63-C
Sec63-N
Sec61α-N
Sec61β-C
F
TMEM 109
TRAPβ
hSnd2
Sec61α
TRAPα
Sec63
Sec61β

**Figure S12. Establishing the interaction between hSnd2 and TMEM109 in living cells by the NanoBiT assay. Related to Figure 5.** (A) To test the most efficient interaction between hSnd2 andTMEM109 six different protein constructs were generated. hSnd2 and TMEM109 are schematically shown in their topology within the ER-membrane. While it was possible to add the SmallBit ($_S$, represented by small light green ball) as well as the LargeBit ($_L$, represented by large dark green sphere) subunit to the N and the C-terminus of hSnd2, it was only possible to tag TMEM109 at the cytosolic C-terminus because of the membrane topology and the presence of a signal peptide at the N-terminus which is cleaved off during biosynthesis. In roman numbers the four possible interactions are indicated. These four combinations were tested with the NanoBiT assay and the results are represented in B. (B) The measurements were normalized to the corresponding negative control, consisting of the Large-Bit subunit and the Halo-Small-Bit construct and are shown in relative light units (RLU). Combination IV show a significantly stronger interaction signal compared to combinations I-III. This laid the foundation for the following experiment where the specificity of this interaction pair IV was proved by a competitive plasmid expression. (C) By a parallel expression of a third plasmid beside the functional combination of TMEM109-$C_L$ and hSnd2-$N_S$ (dark green) the interaction was hampered by an untagged version of hSnd2 (purple) or TMEM109 (blue) or an Empty vector (EV, light green) as control. Again, all samples were normalized against the TMEM109-$C_L$ +Halo-CS control. (D) The correct cytosolic localization of the interacting TMEM109-$C_L$ and hSnd2-$N_S$ constructs were proved by a trypsinization assay. The three lines represent the same combination of proteins but different treatments over time. Dashed timeslots represent timeframes where no measurement was possible due to application of the different components. After the first timepoint the luciferase substrate was added to the cells and an interaction signal emerges. For better comparison the traces were normalized to the timepoint four minutes after substrate application. During the second timepoint Digitonin was added to the wells of the blue and red line, while the black line was treated with DMSO as solvent control. In the third timepoint black and red line were treated with Trypsin, while the blue line was treated with $H_2O$ as control. During the last timepoint, number four, blue line was also treated with Trypsin. By this we were able to show, that the luciferase components were at least cytosolically localized in the cells. Every line represents the mean of a triplicate. (E) Interaction map of multiple tested constructs based on the map published by Sicking et al. [71] and the results that are

shown in Figure 5. The grey boxes represent non interacting combinations while the interaction increases from light green (<25 RLU) to dark green (<100 RLU) and peaks in blue (>100 RLU). Checkmarked interactions were verified by competitive experiments, while crosshatched boxes represent sterically impossible interactions due to reporters on different sides of the membrane. (F) Visualization of the heatmap in an interaction network. Here the thickness of the lines represents the intensity of the measured RLUs. Purple lines show cytosolic and yellow lines represent the luminal interactions. TMEM109 and hSnd2 are integrated into a network which was chracterized in a proof-of-principle study by Sicking et al. [71].

Original Western blots for siRNA mediated silencing shown in Fig. 6A,
where indicated pancreatic rough microsomes (RM) served for identification,
guided by prestained molecular mass markers the PVDF membrane was cut into pieces.

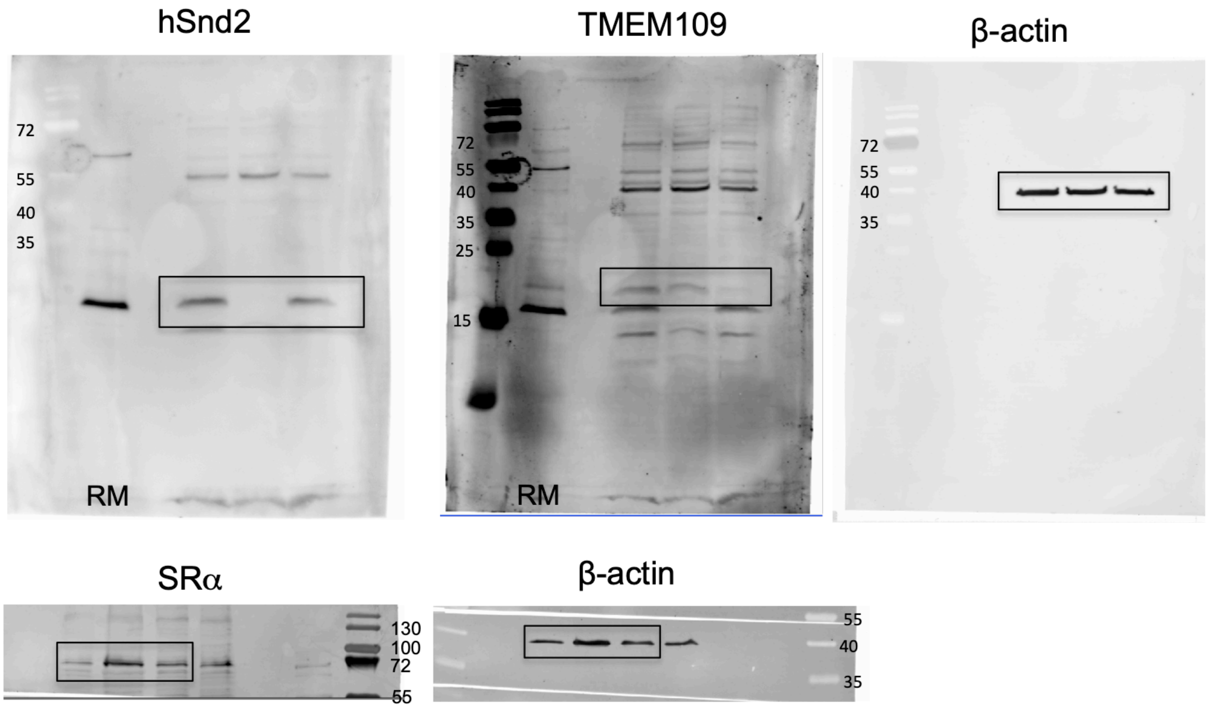

**Figure S13. Original Western blots related to Figure 6A.** Canine pancreatic rough microsomes and molecular mass markers were loaded to the same gels and served for identification of the protein of interest (cropped area).

Original Western blots for siRNA mediated silencing shown in Fig. 6B,
guided by prestained molecular mass markers the two PVDF membranes were cut into pieces.

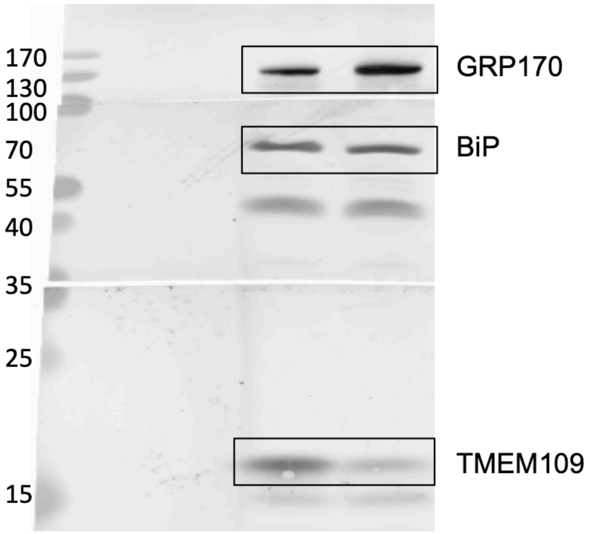


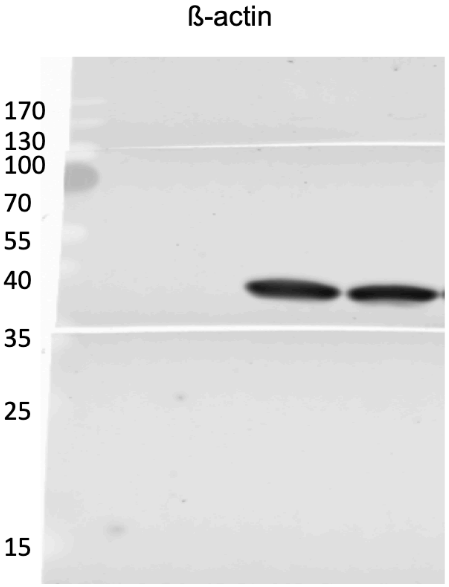


Original Western blots for siRNA mediated silencing shown in Fig. 6B,
where indicated pancreatic rough microsomes (RM) served for identification,
guided by prestained molecular mass markers the two PVDF membranes were cut into pieces.

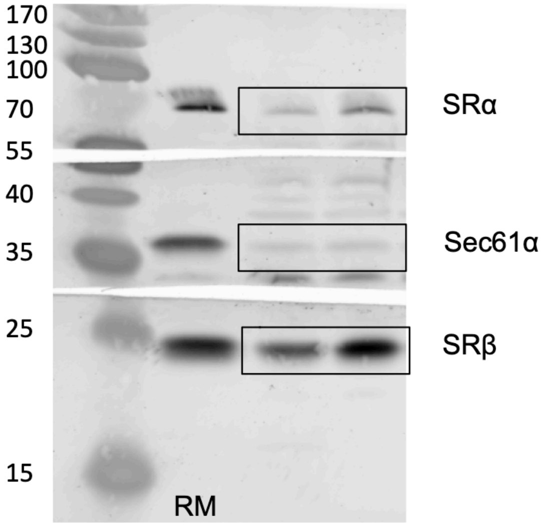


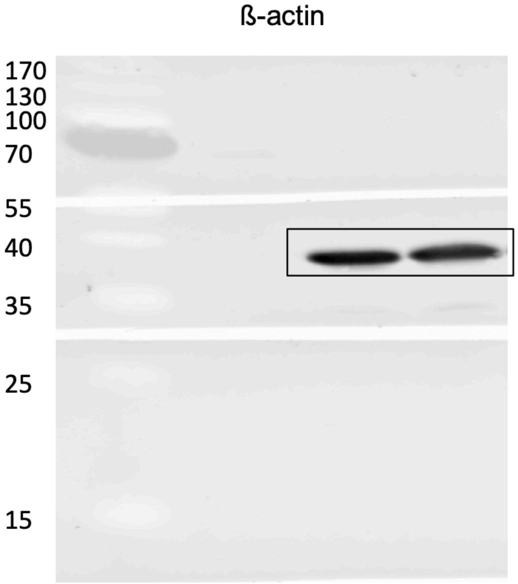

**Figure S14. Original Western blots related to Figure 6B.** Canine pancreatic rough microsomes and molecular mass markers were loaded to the same gels and served for identification of the protein of interest (cropped area).

**A**

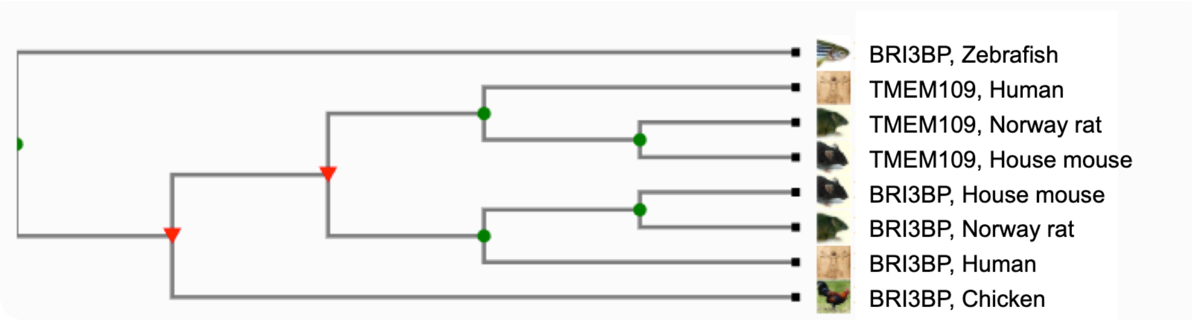


**B**

```
      Signal peptide
1     MAASSISSPWGKHVFKAILMVLVALILLHSALAQSRRDFAPPGQQKREAP      50
      : :     :         :  :  : ::    ::
1     MGARASGGPLARAGLLLLLLLLLLLGLLAPG-AQGARGRGGAEKNSYRRT      49
      Signal peptide
                                       TMD1
51    VDVLTQIGRSVRGTLDAWIGPETMHLVSESSSQVLWAISSAISVAFF--A      100
      :    :   :  :               :              :  :
50    VNTFSQSVSSLFGEDNVRAAQKFLARLTERF---------VLGVDMFVET      90

                                              TMD2
101   LSGIAAQLLNALGLAGDYLAQGLK---LSPGQVQTFLLWGAGALVVYWLLSLLLG      150
             ::  :::    : :       :       ::     :  :: ::: ::
101   LWKVWTELLDVLGLDVSNLSQYFSPASVSSSPARALLLVGVV-LLAYWFLSLTLG      145
                                        TMD1
                                       TMD3
151   LVLALL----GRILWGLKLVIFLAGFVALMRSVPDPSTRALLLLALLI-LYALLS      200
           :    ::  :    :      :              : :      :
151   FTFSVLHVVFGRFFWIVRVVLFSMSCVYILHKYEGEPENAVLPLCFVVAVYFMTG      199
       TMD2                                   TMD3
                        Putative coiled-coil
201   RLT---------GSRASGAQLEAKVRGLERQVEELRWRQRRAAKGARSVEEE      243
                   :  :    : :   :: ::  :  :  :
201   PMGFYWRSSPSGPSNPSNPSVEEKLEHLEKQVRLLNIRLNRVLESLDRSKDK      251
                        Putative coiled-coil
```

**Figure S15. TMEM109 and BRI3BP are part of a protein family. Related to Figure 5.** (A) The phylogenetic tree was generated by the TreeFam option of http://pfam.xfam.org according to Ruan, J. et al. (*Nucleic Acids Res.* **2008**, *36*, D735-40) and Guindon, S. et al. (*Syst Biol,* **2010**, *59*, 307-321). (B) The sequences of human TMEM109 (Q9BVC6) and BRI3BP (Q8WY22) were extracted from UniprotKB. Sequence alignment was performed with the ClustalV option of the MegAlign tool within the DNASTAR sofware package (Lasergene 12). Signal peptides, transmembrane domains (TMD) and coiled coil domains are indicated as determined by UniProtKB, https://services.healthtech.dtu.dk/service.php?SignalP and http://gpcr.biocomp.unibo.it/cgi/predictors/cc/pred_cchmm.cgi, respectively.

**Table S1, related to Figure 1A.**

File Name: Table S1_sra_all.xlsx

Complete list of genes corresponding to proteins quantified after *SRA* silencing in HeLa cells. Gene names, protein accession numbers (ID), log2 fold changes resulting from siRNA-mediated SR$\alpha$ depletion, and -log10 p values are indicated. Minus sign in front of fold change denotes negatively affected proteins. The number of listed proteins differs from the total number of quantified proteins because some proteins were quantified in less than two of the triplicates. The original Orbitrap data for all quantified proteins are deposited at Proteome Exchange: http://www.proteomexchange.org.

**Table S2, related to Figure 1A.**

File Name: Table S2_sra_full_lo.xlsx

Proteins that were negatively affected by *SRA* silencing in HeLa cells, i.e. putative SR$\alpha$ clients. Gene names, protein accession numbers (ID), and log2 fold changes resulting from siRNA-mediated SR$\alpha$ depletion are presented together with full protein names and Gene Ontology (GO) annotations for subcellular location(s), presence of N-terminal signal peptide (SP) or most N-terminal transmembrane helix (TMH), number of N-glycosylation sites (Glycosylation sites), number of transmembrane domains (TMD), amino acid sequences of SP or TMH (in single letter code), position of TMH, all as extracted from UniProtKB entries using custom scripts. Proteins are listed according to decreasing negative effects of SR$\alpha$ depletion.

**Table S3, related to Figure 1A.**

File Name: Table S3_sra_full_up.xlsx

Proteins that were positively affected by *SRA* silencing in HeLa cells. Gene names, protein accession numbers, and log2 fold changes resulting from SR$\alpha$ depletion are presented together with Gene Ontology (GO) annotations for subcellular location(s), presence of N-terminal signal peptide (SP) or N-terminal transmembrane helix (TMH), number of N-glycosylation sites (Glycosylation sites), amino acid sequences of SP or TMH (in single letter code), all as extracted from UniProtKB entries using custom scripts. Proteins are listed according to decreasing positive effects of SR$\alpha$ depletion.

**Table S4, related to Figure 2. Summary of clients as determined by MS and differential protein abundance analysis.**

| Clients of | Clients with | | | Clients with | | | | |
|---|---|---|---|---|---|---|---|---|
| | SP | SP/I | SP/multi | TMH | TMH/II or III | TMH/multi | HP | TA |
| **SRα** | **24** | **14** | **-** | **30** | **10** | **17** | **3** | **-** |
| Total | 54 | | | | | | | |
| % | 44.4 SP | | | 55.6 TMH | | | | |
| % | 81.5 MP | | | | | | | |
| % of TMH | | | | | 33.3 | 56.7 | 10 | 0 |
| % | 18.5sol | 25.9 | | | 18.5 | 31.5 | 5.6 | 0 |
| **Wrb** | **13** | **6** | **-** | **14** | **2** | **8** | **1** | **3** |
| Total | 27 | | | | | | | |
| % | 48.1 SP | | | 51.9 TMH | | | | |
| % | 74.1 MP | | | | | | | |
| % of TMH | | | | | 14.3 | 57.1 | 7.1 | 21.4 |
| % | | | | | 7.4 | 29.6 | 3.7 | 10.1 |
| **hSnd2** | **3** | **3** | **-** | **9** | **2** | **5** | **-** | **2** |
| Total | 12 | | | | | | | |
| % | 25 SP | | | 75 TMH | | | | |
| % | 100 MP | | | | | | | |
| % of TMH | | | | | 22.2 | 55.6 | 0 | 22.2 |
| % | | | | | 16.7 | 41.7 | 0 | 16,7 |
| **hSnd2+Wrb** | **13** | **5** | **2** | **30** | **3** | **21** | **1** | **5** |
| Total | 43 | | | | | | | |
| % | 30.2 SP | | | 69.8 TMH | | | | |
| % | 86 MP | | | | | | | |
| % of TMH | | | | | 10 | 70 | 3.3 | 16.7 |
| % | | | | | 7 | 48.8 | 2.3 | 11.6 |
| **Wrb** sum | **26** | **11** | **2** | **44** | **5** | **29** | **2** | **8** |
| Total | 70 | | | | | | | |
| % | 37.1 SP | | | 62.9 TMH | | | | |
| % | 81.4 MP | | | | | | | |
| % of TMH | | | | | 11.4 | 65.9 | 4.5 | 18.2 |
| % | 18.6sol | 15.7 | 2.9 | | 7.1 | 41.4 | 2.9 | 11.4 |
| **hSnd2** sum | **16** | **8** | **2** | **39** | **5** | **26** | **1** | **7** |
| Total | 55 | | | | | | | |
| % | 29,1 SP | | | 70.9 TMH | | | | |
| % | 89.1 MP | | | | | | | |
| % of TMH | | | | | 12.8 | 66.7 | 2.6 | 17.9 |
| % | 10.9sol | 14.5 | 3.6 | | 9.1 | 47.3 | 1.8 | 12.7 |
| **PEX3** | 1 | - | 1 | 2 | 1 | 1 | - | - |
| Zellweger | 27 | 5 | - | 20 | 8 | 6 | 2 | 4 |
| | **28** | **5** | **1** | **22** | **9** | **7** | **2** | **4** |
| Total | 50 | | | | | | | |
| % | 56 SP | | | 44 TMH | | | | |
| % | 56 MP | | | | | | | |
| % of TMH | | | | | 40.9 | 31.8 | 9.1 | 18.2 |
| % | 44sol | 10 | 2 | | 18 | 14 | 4 | 8 |

I, II, III, membrane protein type; HP, hairpin; MP, membrane protein; multi, multispanning membrane protein; sol, soluble; SP, signal peptide; sum, sum of clients as determined in single depletion and double depletion (hSnd2+Wrb); TA, tail anchor; TMH, most N-terminal transmembrane domain; Zellweger, data from PEX3 deficient patient fibroblasts [38]. Notably, sum refers to the pool of the respective single depletion (hSnd2 or Wrb) and the double depletion (hSnd2+Wrb).

**Table S5, related to Figure 2. Characteristics of clients of SRα, Wrb, and hSnd2 with TMH.**

| | TMDs | Type | ΔGapp | Sequence | 1st TMH | Size | % | N-glyco |
|---|---|---|---|---|---|---|---|---|
| **SRα** | | | | | | | | |
| ABCC4 | multi | | 5.118 | LVLGIFTLIEESAKVIQPIFL | 93-113 | 1325 | 7.8 | + |
| ANO10 | multi | | 1.133 | IALYFGFLEYFTFALIPMAVI | 208-228 | 660 | 32.7 | |
| ASPH | 1 | II | -1.493 | FFTWFMVIALLGVWTSVAVVW | 54-74 | 758 | 8.4 | + |
| ATL2 | 1 | HP | -0.074 | TLFAVMFAMYIISGLTGFIGL | 477-497 | 583 | 83.5 | + |
| ATP2B1 | multi | | 4.019 | FLQLVWEALQDVTLIILEIAA | 98-118 | 1220 | 8.9 | |
| ATP13A1 | multi | | 1.928 | VLPFAGLLYPAWLGAAAAGCW | 67-87 | 1204 | 6.4 | + |
| B3GALTL | 1 | II | -0.935 | WWLLAPPALLALLTCSLAFGL | 7-27 | 498 | 3.4 | + |
| BST2 | 1 | II | -5.685 | KLLLGIGILVLLIIVILGVPLIIFTIKA | 21-48 | 180 | 17.2 | + |
| CAV1 | 1 | HP | -0.726 | ALFGIPMALIWGIYFAILSFL | 105-125 | 178 | 64.4 | |
| CEPT1 | multi | | -0.476 | LITIIGLSINICTTILLVFYC | 87-107 | 416 | 23.3 | + |
| DEGS1 | multi | | -0.478 | PNLIWIIIMMVLTQLGAFYIV | 41-61 | 323 | 15.8 | |
| ERGIC2 | multi | | 1.141 | GTVSLIAFTTMALLTIMEFSV | 34-54 | 377 | 11.7 | |
| ERLIN2 | 1 | II | 1.947 | LGAVVAVASSFFCASLFSAVH | 4-24 | 339 | 4.1 | + |
| IKBIP | 1 | | 0.469 | CLSLLSLGTCLGLAWFV | 46-62 | 377 | 14.9 | + |
| ITPR3 | multi | | 0.138 | LWGSISFNLAVFINIIIAFFY | 2203-2223 | 2671 | 82.9 | + |
| LNPEP | 1 | II | -2.599 | MVVCAFVIVVAVSVIMVIYLL | 111-131 | 1025 | 11.8 | |
| PDE3A | multi | | -0.598 | LSSALCAGSLSFLLALLVRLV | 61-81 | 1141 | 6.2 | |
| PEX3 | 1 | | 3.594 | CIFLGTVLGGVYILGKYGQKK | 16-36 | 373 | 7 | |
| REEP3 | multi | HP | 0.697 | MVSWMISRAVVLVFGMLYPAY | 1-21 | 255 | 4.3 | |
| SLC16A3 | multi | | -0.663 | GGWGWAVLFGCFVITGFSYAF | 18-38 | 465 | 6 | |
| SLC35B2 | multi | | 3.884 | WWAVVVLAAFPSLGAGGETPE | 5-25 | 432 | 3.5 | |
| SPTLC1 | 1 | | 1.444 | ALYEAPAYHLILEGILILWII | 16-36 | 513 | 5.1 | |
| SUN1 | 1 | II | -1.860 | ICKFLVLLIPLFLLLAGLSL | 316-335 | 785 | 41.5 | |
| TMEM41B | multi | | -1.951 | MSLLILVSIFLSAAFVMFLVY | 52-72 | 291 | 21.3 | |
| TMEM209 | multi | | 1.532 | VVLAWGLLNVSMAGMIYTEMT | 28-48 | 561 | 6.8 | + |
| TMTC3 | multi | | 1.675 | ITLIVGVVTACYWNSLFCGFV | 9-29 | 914 | 1.8 | + |
| TOR1AIP2 | 1 | | -0.371 | FWSYGPVILVVLVVAVVASSV | 215-235 | 470 | 47.9 | + |
| TVP23B | multi | | 1.886 | PVASFFHLFFRVSAIIVYLL | 34-53 | 205 | 21.5 | |
| YIPF5 | multi | | 0.365 | TDLAGPMVFCLAFGATLLLAG | 125-145 | 257 | 52.5 | |
| ZMPSTE24 | multi | | 1.532 | IFGAVLLFSWTVYLWETFLAQ | 19-39 | 475 | 6.1 | |
| **Wrb** | | | | | | | | |
| EXT2 | 1 | II | -1.428 | YITLFSIVLLGLIATGMFQFW | 26-46 | 718 | 5 | |
| FAR1 | 1 | TA | -0.255 | IRYGFNTILVILIWRIF | 466-483 | 515 | 92.4 | |
| GOLGA5 | 1 | TA | -2.261 | VFVIIYMALLHLWVMIVLLTYTP | 699-719 | 731 | 97 | |
| ITPR3 | multi | | 0.138 | LWGSISFNLAVFINIIIAFFY | 2203-2223 | 2671 | 82.9 | |
| LBR | multi | | -2.378 | VPGVFLIMFGLPVFLFLLLLM | 212-232 | 615 | 36.1 | |
| MARCH1 | multi | | 0.107 | IFCSVTFHVIAITCVVWSLYV | 155-175 | 545 | 30.3 | |
| MFSD7 | multi | | 0.335 | WVFLLAISLLNCSNATLWLSF | 30-50 | 559 | 7.2 | |
| MFSD10 | multi | | -1.459 | VVFLGLLLDLLAFTLLLPLLP | 27-47 | 455 | 8.1 | |
| NEU1 | 1 | | -1.467 | LGFWGGCRVWVFAAIFLLLSLAA | 20-41 | 415 | 7.2 | + |
| PTDSS1 | multi | | 0.972 | FFYRPHTITLLSFTIVSLMYF | 36-56 | 473 | 9.7 | |
| REEP3 | multi | HP | 0.697 | MVSWMISRAVVLVFGMLYPAY | 1-21 | 255 | 4.3 | |
| SGPP1 | multi | | 2.661 | FCFGTELGNELFYILFFPFWI | 132-152 | 441 | 32.2 | |
| SPCS2 | multi | | 1.255 | ICTISCFFAIVALIWDYMHPF | 87-107 | 226 | 82.9 | |
| UBE2J2 | 1 | TA | -1.019 | GLLGGALANLFVIVGFAAFAYTW | 227-247 | 259 | 91.5 | |
| **hSnd2** | | | | | | | | |
| MYO9A | 1 | | 1.571 | IYTYVGSILIVINPFKFLPIY | 175-195 | 2548 | 7.3 | |
| PTDSS2 | multi | | -0.192 | AHTLTVLFILTCTLGYVTLLE | 63-83 | 487 | 15 | + |
| PTGIS | 1 | | -3.435 | MAWAALLGLLAALLLLLLS | 1-20 | 500 | 2.2 | |
| SLC4A2 | multi | | -0.458 | CLAAVIFIYFAALSPAITFGGLLG | 708-731 | 1241 | 57.9 | + |
| SLC7A2 | multi | | 2.693 | DLIALGVGSTLGAGVYVLAGEV | 38-59 | 658 | 7.3 | + |
| TMEM41B | multi | | -1.951 | MSLLILVSIFLSAAFVMFLVY | 52-72 | 291 | 21.3 | |
| TRPM7 | multi | | 1.975 | NSWYKVILSILVPPAILLLEY | 756-776 | 1865 | 41.1 | |
| VAMP4 | 1 | TA | -3.47 | IKAIMALVAAILLLVIIILIV | 116-136 | 141 | 89.4 | |
| VAMP8 | 1 | TA | -4.429 | MVLICVIVFIIILFIVLFAT | 76-96 | 100 | 86 | |

1st TMH, most N-terminal transmembrane domain; TMD, number of transmembrane domains (including TMH); 1, single spanning; multi, multispanning; Type, membrane protein type; HP, hairpin; TA, tail anchor; ΔGapp, apparent delta G of TMH; Sequence, primary structure of TMH; amino acid residues of TMH; Size, number of amino acid residues of client; %, distribution of TMH in client (i.e. position of central amino acid residue of TMH in % of client); N-glyco, N-glycosylation.

**Table S6, related to Figure 2. Characteristics of clients of Wrb plus hSnd2 and PEX3 with TMH.**

| | TMDs | Type | ΔGapp | Sequence | TMH | Size | % | N-glyco |
|---|---|---|---|---|---|---|---|---|
| **hSnd2+Wrb** | | | | | | | | |
| AGPAT5 | multi | | 1.565 | LLPSVVLLGTAPTYVLAWGVW | 15-35 | 364 | 6.9 | |
| ATG9A | multi | | -0.147 | IFELMQFLFVVAFTTFLVSCV | 67-87 | 839 | 9.2 | + |
| ATP2C1 | multi | | 4.913 | LWKKYISQFKNPLIMLLLASA | 71-91 | 919 | 8.8 | |
| ATP12A | multi | | 3.561 | EIVKFLKQMVGGFSILLWVGA | 102-123 | 1093 | 10.3 | |
| BCAP29 | multi | | -0.863 | AVATFLYAEIGLILIFCLPFI | 7-27 | 241 | 7.1 | |
| C4orf3 | 1 | TA | -2.704 | SYWLDLWLFILFDVVVFLFVYFL | 45-65 | 65 | 84.6 | |
| CXCR4 | multi | | -0.37 | IFLPTIYSIIFLTGIVGNGLVILVM | 39-63 | 352 | 13.9 | + |
| EMD | 1 | TA | -2.850 | VPLWGQLLLFLVFVIVLFFIY | 223-243 | 254 | 91.7 | |
| GDPD4 | multi | | 0.185 | WVTFLGTGYWFFWSIFILSLA | 18-38 | 520 | 5.4 | + |
| JPH1 | 1 | TA | -1.838 | IMIVLVMLLNIGLAILFVHFL | 640-660 | 661 | 98.3 | |
| LEMD2 | multi | | -3.464 | LLLWASLGLLLVFLGILWVKM | 213-233 | 503 | 44.3 | |
| MBOAT7 | multi | | 2.483 | LVVLLISIPIGFLFKKAGPGL | 9-29 | 472 | 4 | + |
| MXRA7 | 1 | | 2.128 | LLAALPALATALALLLAWLLV | 7-27 | 170 | 10 | |
| PLD3 | 1 | II | -1.979 | VLLVLILAVVGFGALMTQLFL | 39-59 | 490 | 10 | + |
| POMK | 1 | II | -0.095 | VGLLLIMALMNTLLYLCLDHFFI | 21-43 | 350 | 8.9 | + |
| PRAF2 | multi | | 0.209 | LYYQTNYLLCFGIGLALAGYV | 42-62 | 178 | 29.2 | |
| RHBDD2 | multi | | 2.164 | WCLCPEVPSATFFTALLSLLV | 11-31 | 364 | 5.8 | |
| REEP5 | multi | HP | -1.133 | SFIALGVIGLVALYLVFGYGA | 35-55 | 189 | 23.8 | |
| SEC62 | multi | | -1.555 | FVMGLILVIAVIAATLFPLWP | 197-217 | 399 | 51.9 | |
| SLC9A6 | multi | | 3.829 | LWLLLAVGVFDWAGASDGGGG | 28-48 | 679 | 5.6 | + |
| SLC16A7 | multi | | -0.035 | GGWGWIVVGAAFISIGFSYAF | 16-36 | 478 | 5.4 | |
| SLC39A7 | multi | | 0.193 | WVAVGLLTWATLGLLVAGLGG | 10-30 | 469 | 4.3 | |
| SOAT1 | multi | | -2.080 | IYHMFIALLILFILSTLVV | 141-159 | 550 | 27.5 | |
| STEAP4 | multi | | -0.321 | LFPMWRFPFYLSAVLCVFLFF | 196-216 | 459 | 44.9 | |
| STX2 | 1 | TA | -2.654 | WIIIAVSVVLVAIIALIIGLSVGK | 265-288 | 288 | 95.5 | |
| STX3 | 1 | TA | -4.337 | LIIIIVLVVVLLGILALIIGLSV | 264-284 | 289 | 94.8 | |
| STX17 | multi | | -2.116 | LAALPVAGALIGGMVGGPIGL | 229-249 | 302 | 79.1 | |
| TMEM33 | multi | | 0.388 | LFTVYCSALFVLPLLGLHEAA | 32-52 | 247 | 17 | + |
| TMEM38B | multi | | 0.584 | SWFTAMLHCFGGGILSCLLLA | 50-70 | 291 | 20.6 | |
| TMEM181 | multi | | -2.195 | HFVLVFVVFFICFGLTIFVGI | 153-173 | 475 | 34.4 | |
| | | | | | | | | |
| **PEX3** | | | | | | | | |
| ABCD3 | multi | | 1.380 | GYLVLIAVMLVSRTYCDVWMI | 84-104 | 659 | 14.3 | + |
| ACBD5 | 1 | | -0.121 | GVLTFAIIWPFIAQWLVYLYY | 497-517 | 525 | 96.6 | |
| AIFM2 | 1 | | 2.886 | VESGALHVVIVGGGFGGIAAA | 7-27 | 373 | 4.6 | |
| ATL1 | 1 | HP | -0.573 | TLFVVIFITYVIAGVTGFIGL | 450-470 | 558 | 82.4 | |
| CCDC136 | 1 | TA | -2.371 | IFSLPLVGLVVISALLWCWWA | 1130-1150 | 1154 | 98.8 | |
| COLEC12 | 1 | II | -2.162 | FSIILLYILCALLTITVAILG | 38-58 | 742 | 6.5 | + |
| CYBRD1 | multi | | -2.348 | LLGSALLVGFLSVIFALVWVL | 12-32 | 286 | 7.7 | + |
| DHRS7B | 1 | II | -0.36 | FITSTAILPLLFGCLGVFGLF | 18-38 | 325 | 8.6 | |
| ENPP1 | 1 | II | -0.985 | VLSLVLSVCVLTTILGCIFGL | 77-97 | 925 | 9.4 | + |
| ERMP1 | multi | | 2.156 | AGTGLSEVRAALGLALYLIAL | 64-84 | 904 | 8.2 | + |
| FAR1 | 1 | TA | -0.668 | IRYGFNTILVILIWRIFI | 466-483 | 515 | 92.4 | |
| MAN1A1 | 1 | II | -2.612 | FVLLLVFSAFITLCFGAIFFL | 42-62 | 653 | 8 | + |
| PEX13 | 1 | | 0.039 | AATSAKSWPIFLFFAVILGGPYLIW | 227-251 | 403 | 58.8 | |
| PXMP2 | multi | | 3.630 | LYPVLTKAATSGILSALGNFL | 31-51 | 195 | 21 | |
| RTN3 | multi | HP | -2.436 | LIMLLSLAAFSVISVVSYLILALL | 864-887 | 1032 | 84.7 | |
| SGCD | 1 | II | -3.808 | FFVLLLMILILVNLAMTIWIL | 37-57 | 256 | 18.4 | |
| STX6 | 1 | TA | -5.096 | WCAIAILFAVLLVVLILFLVL | 235-255 | 255 | 96.1 | + |
| TMEM192 | multi | | -1.154 | TVIIVNLLWFIHLVFVVLAFL | 47-67 | 271 | 21 | |
| TMEM237 | multi | | 1.917 | MIGLFSHGFLAGCAVWNIVVI | 227-247 | 408 | 58.1 | |
| TMUB2 | multi | | -2.382 | VMVVAGVVVLILALVLAWLST | 36-56 | 321 | 14.3 | |
| TOR1AIP1 | 1 | | 0.705 | WLLPLIAALASGSFWFF | 339-355 | 583 | 59.9 | |
| VAMP3 | 1 | TA | -4.236 | MWAIGITVLVIFIIIIIVWVV | 78-98 | 100 | 88 | |

1st TMH, most N-terminal transmembrane domain; TMD, number of transmembrane domains (including TMH); 1, single spanning; multi, multispanning; Type, membrane protein type; HP, hairpin; TA, tail anchor; ΔGapp, apparent delta G of TMH; Sequence, primary structure of TMH; amino acid residues of TMH; Size, number of amino acid residues of client; %, distribution of TMH in client (i.e. position of central amino acid residue of TMH in % of client); N-glyco, N-glycosylation.

**Table S7, related to Figure 1B.**

File Name: Table S7_wrb_all.xlsx

Complete list of genes corresponding to proteins quantified after *WRB* silencing in HeLa cells. Gene names, protein accession numbers (ID), log2 fold changes resulting from siRNA-mediated Wrb depletion, and -log10 p values are indicated. Minus sign in front of fold change denotes negatively affected proteins. The original Orbitrap data for all quantified proteins are deposited at Proteome Exchange: http://www.proteomexchange.org.

**Table S8, related to Figure 1B.**

File Name: Table S8_wrb_full_lo.xlsx

Proteins that were negatively affected by *WRB* silencing in HeLa cells, i.e. putative Wrb clients. Gene names, protein accession numbers (ID), and log2 fold changes resulting from siRNA-mediated Wrb depletion are presented together with full protein names and Gene Ontology (GO) annotations for subcellular location(s), presence of N-terminal signal peptide (SP) or most N-terminal transmembrane helix (TMH), number of N-glycosylation sites (Glycosylation sites), number of transmembrane domains (TMD), amino acid sequences of SP or TMH (in single letter code), position of TMH, all as extracted from UniProtKB entries using custom scripts. Proteins are listed according to decreasing negative effects of Wrb depletion.

**Table S9, related to Figure 1B.**

File Name: Table S9_wrb_full_up.xlsx

Proteins that were positively affected by *WRB* silencing in HeLa cells. Gene names, protein accession numbers, and log2 fold changes resulting from Wrb depletion are presented together with Gene Ontology (GO) annotations for subcellular location(s), presence of N-terminal signal peptide (SP) or N-terminal transmembrane helix (TMH), number of N-glycosylation sites (Glycosylation sites), amino acid sequences of SP or TMH (in single letter code), all as extracted from UniProtKB entries using custom scripts. Proteins are listed according to decreasing positive effects of Wrb depletion.

**Table S10, related to Figure 3A.**

File Name: Table S10_snd_all.xlsx

Complete list of genes corresponding to proteins quantified after *hSND2* silencing in HeLa cells. Gene names, protein accession numbers (ID), log2 fold changes resulting from siRNA-mediated hSnd2 depletion, and -log10 p values are indicated. Minus sign in front of fold change denotes negatively affected proteins. The original Orbitrap data for all quantified proteins are deposited at Proteome Exchange: http://www.proteomexchange.org.

**Table S11, related to Figure 3A.**

File Name: Table S11_snd_full_lo.xlsx

Proteins that were negatively affected by *hSND2* silencing in HeLa cells, i.e. putative hSND2 clients. Gene names, protein accession numbers (ID), and log2 fold changes resulting from siRNA-mediated hSnd2 depletion are presented together with full protein names and Gene Ontology (GO) annotations for subcellular location(s), presence of N-terminal signal peptide (SP) or most N-terminal transmembrane helix (TMH), number of N-glycosylation sites (Glycosylation sites), number of transmembrane domains (TMD), amino acid sequences of SP or TMH (in single letter code), position of TMH, all as extracted from UniProtKB entries using custom scripts. Proteins are listed according to decreasing negative effects of hSnd2 depletion.

**Table S12, related to Figure 3A.**

File Name: Table S12_snd_full_up.xlsx

Proteins that were positively affected by *hSND2* silencing in HeLa cells. Gene names, protein accession numbers, and log2 fold changes resulting from hSnd2 depletion are presented together with Gene Ontology (GO) annotations for subcellular location(s), presence of N-terminal signal peptide (SP) or N-terminal transmembrane helix (TMH), number of N-glycosylation sites (Glycosylation sites), amino acid sequences of SP or TMH (in single letter code), all as extracted from UniProtKB entries using custom scripts. Proteins are listed according to decreasing positive effects of hSnd2 depletion.

**Table S13, related to Figure 3B.**

File Name: Table S13_sw_all.xlsx

Complete list of genes corresponding to proteins quantified after simultaneous *hSND2* and *WRB* silencing in HeLa cells. Gene names, protein accession numbers (ID), log2 fold changes resulting from siRNA-mediated hSnd2 and Wrb depletion, and -log10 p values are indicated. Minus sign in front of fold change denotes negatively affected proteins. The original Orbitrap data for all quantified proteins are deposited at Proteome Exchange: http://www.proteomexchange.org.

**Table S14, related to Figure 3B.**

File Name: Table S14_sw_full_lo.xlsx

Proteins that were negatively affected by simultaneous *hSND2* and *WRB* silencing in HeLa cells, i.e. putative hSnd2 and Wrb clients. Gene names, protein accession numbers (ID), and log2 fold changes resulting from siRNA-mediated hSnd2 and Wrb depletion are presented together with full protein names and Gene Ontology (GO) annotations for subcellular location(s), presence of N-terminal signal peptide (SP) or most N-terminal transmembrane helix (TMH), number of N-glycosylation sites (Glycosylation sites), number of transmembrane domains (TMD), amino acid sequences of SP or TMH (in single letter code), position of TMH, all as extracted from UniProtKB entries using custom scripts. Proteins are listed according to decreasing negative effects of hSnd2 and Wrb depletion.

**Table S15, related to Figure 3B.**

File Name: Table S15_sw_full_up.xlsx

Proteins that were positively affected by simultaneous *hSND2* and *WRB* silencing in HeLa cells. Gene names, protein accession numbers, and log2 fold changes resulting from hSnd2 and Wrb depletion are presented together with Gene Ontology (GO) annotations for subcellular location(s), presence of N-terminal signal peptide (SP) or N-terminal transmembrane helix (TMH), number of N-glycosylation sites (Glycosylation sites), amino acid sequences of SP or TMH (in single letter code), all as extracted from UniProtKB entries using custom scripts. Proteins are listed according to decreasing positive effects of hSnd2 and Wrb depletion.

**Table S16. Related to Figure 5.**

File Name: Table S16_Overlap siSND, siSND+WRB, CO-IP hSnd2.xlsx

**Table S17. ProteomeXchange_identifiers**

| | | |
|---|---|---|
| Orbi1986 | SRA experiment 1 | PXD008178 |
| Sample 1-3 | scr control siRNA | |
| Sample 4-6 | SRA siRNA #3 | |
| Sample 7-9 | SRA siRNA #6 | |
| | | |
| Orbi2048 | WRB | PXD008178 |
| Sample 16-18 | scr control siRNA | |
| Sample 19-21 | WRB siRNA #3 | |
| Sample 22-24 | WRB siRNA #4 | |
| | | |
| Orbi2155 | SRA experiment 2 | PXD012078 |
| Sample 1-3 | scr control siRNA | |
| Sample 10-12 | SRA siRNA #3 | |
| Sample 13-15 | SRA siRNA #6 | |
| | | |
| Orbi2514 | SND2 & WRB+SND2 experiment 2 | PXD011993 |
| Sample 1-3 | scr control siRNA | |
| Sample 4-6 | SND2 siRNA #2 | |
| Sample 7-9 | SND2 siRNA #3 | |
| Sample 10-12 | WRB siRNA #3 + SND2 siRNA #2 = SW | |
| Sample 13-15 | WRB siRNA #3 + SND2 siRNA #3 = SW | |

The mass spectrometry proteomics data (.raw and .txt files) have been deposited to the ProteomeXchange Consortium via the PRIDE partner repository with the dataset identifiers: PXD008178, PXD011993 and PXD012078 (http://www.proteomexchange.org).
